# Efficacy and safety of ultraviolet C therapy for the eradication of *Helicobacter pylori*

**DOI:** 10.1186/s12876-025-04471-0

**Published:** 2025-12-10

**Authors:** Ming’e Wu, Yan Li, Guxiang Zhou, Zhenyu Li, Rongxin He, Qingqing Yang, Mengyun Ke, Nana Zhang, Xiaoyan Zeng, Yi Lyu

**Affiliations:** 1 National Local Joint Engineering Research Center for Precision Surgery & Regenerative Medicine, the First Affiliated Hospital of Xi’an Jiaotong University, Xi’an, 710061 China; 2 Shaanxi Province Center for Regenerative Medicine and Surgery Engineering Research, the First Affiliated Hospital of Xi’an Jiaotong University, Xi’an, 710061 China; 3 Department of Clinical Laboratory, Microbiology laboratory, the First Affiliated Hospital of Xi’an Jiaotong University, Xi’an, Shaanxi Province 710061 China

**Keywords:** Helicobacter pylori, Therapy, Irradiation, UVC-LED༛Gastric mucosa, DNA damage

## Abstract

**Background:**

*Helicobacter pylori* (Hp) is a globally prevalent gastrointestinal pathogen and a major etiological agent in various digestive tract disorders. Ultra violet C (UVC) light represents a promising physical modality for the treatment of Hp infection, owing to its capacity for non-invasive endoscopic delivery to eradicate the bacterium within the gastric environment.

**Objective:**

The purpose of this study was to investigate the efficacy and safety of UVC-LED (254/265/275 nm) in the treatment of Hp, both in *vitro* and in *vivo*.

**Methods:**

The bactericidal effect of UVC-LED irradiation against *H. pylori* was assessed in vitro and in an animal infection model. Gastric tissue damage and therapeutic efficacy were evaluated via H&E, γ-H2AX, TUNEL staining and digestive function of the stomach.

**Results:**

UVC-LEDs emitting at 254, 265, and 275 nm exhibited significant anti-*H. pylori* activity. However, a clear divergence in safety profiles was observed. The 275 nm wavelength achieved substantial bacterial load reduction while inducing minimal DNA damage and cellular apoptosis, as evidenced by significantly lower levels of γ-H2AX and TUNEL positivity compared to the 254 nm and 265 nm group at the critical 1-day and 3-day timepoints (*p* < 0.05). Histological analysis confirmed that this attenuated cellular damage correlated with preserved mucosal architecture in the 275 nm treatment group. Furthermore, none of the three wavelengths showed any significant adverse impact on gastric digestive function.

**Conclusions:**

The 275 nm UVC-LED emerges as an effective and tissue-compatible physical therapy for *H. pylori* infection, combining robust bactericidal activity with enhanced mucosal safety. This modality shows promise as a novel complementary approach to conventional antibiotic-based regimens.

**Graphical Abstract:**

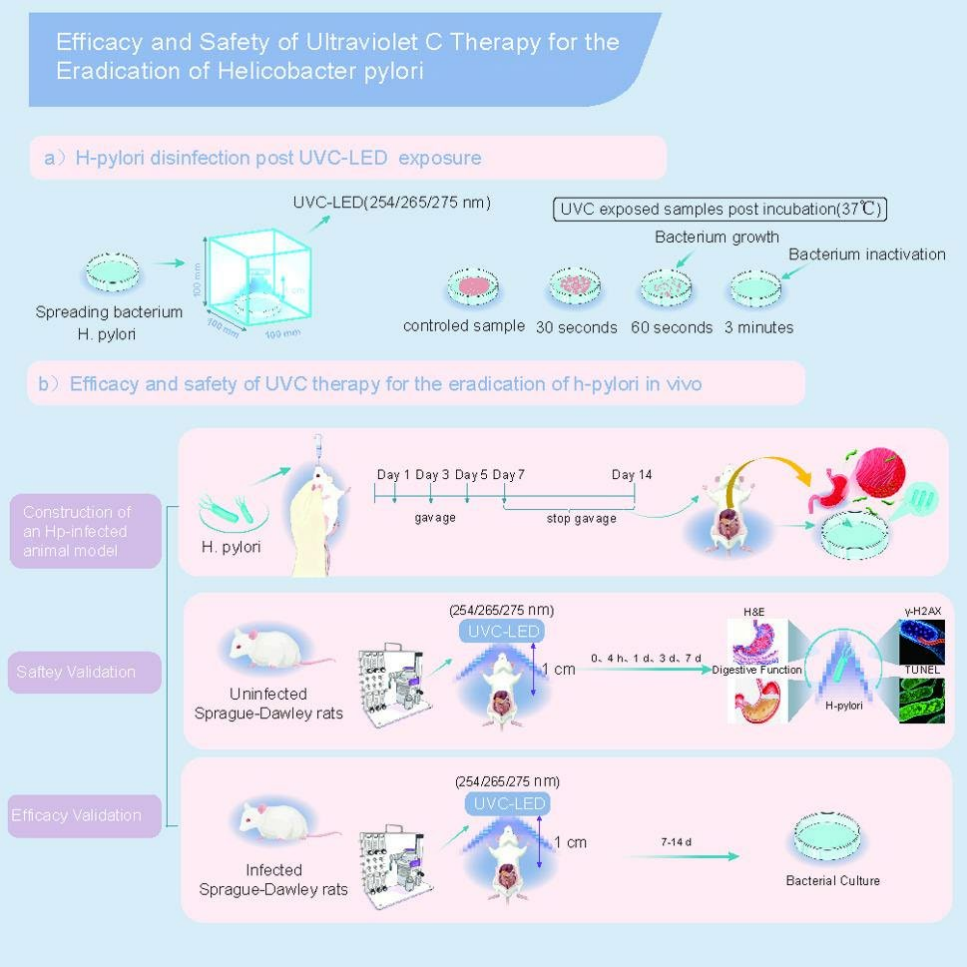

## Background

*Helicobacter pylori* (Hp) is a Gram-negative pathogen transmitted through fecal-oral or oral-oral routes, with a global infection rate of about 43.1% in the last ten years [1]. The infection is typically contracted during early childhood, resulting in a persistent and progressive form of gastritis that can lead to clinical complications in 1–10% of those infected. These complications may include peptic ulcer disease, gastric atrophy, gastric intestinal metaplasia, and, in severe cases, gastric cancer or mucosa-associated lymphoid tissue (MALT) lymphoma [2]. While the prevalence of Hp infection within the population demonstrates a declining trajectory, the emergence of antibiotic resistance in Hp strains presents a considerable challenge to global public health [3]. In the Asia-Pacific region, the resistance rates to commonly utilized anti-Hp medications, including clarithromycin, metronidazole, and levofloxacin, have already attained levels of 30%, 60%, and 35%, respectively [4]. Moreover, the mechanisms underlying antibiotic resistance in Hp are complex and encompass various forms, including mono-resistance, multidrug resistance, and heteroresistance [5, 6]. Furthermore, the misuse of antibiotics can result in the emergence of drug-resistant subpopulations that may exist prior to the commencement of antimicrobial therapy. This phenomenon can contribute to recurrent infections and potentially exacerbate the development of resistance [7]. Challenges persist due to the restricted treatment options for Hp and the increased antibiotic resistance associated with this pathogen. Consequently, there is an urgent need for the development of innovative antibacterial therapies targeting Hp to enhance the cure rates associated with this infection.

In recent years, ultraviolet (UV) disinfection has gained increasing popularity as an innovative disinfection technology, particularly due to its rapid disinfection capabilities and the absence of secondary pollution [8]. Ultraviolet radiation can be classified into three distinct categories based on wavelength: ultraviolet-A band (UVA) (400–320 nm), ultraviolet-B band (UVB) (320–280 nm), and ultraviolet-C band (UVC) (280–100 nm) [9]. Owing to their compact size, energy efficiency, and durability, UVC LEDs provide a chemical-free and highly effective method for reducing microbial contamination. This has paved the way for their widespread use in ensuring cleaner environments across multiple sectors, including healthcare, water and air purification, and surface disinfection.

UVC radiation corresponds with the absorption spectra of DNA, RNA, and proteins. This radiation has the potential to cause damage to DNA by facilitating the formation *thymine dimers* (specifically cyclobutane pyrimidine dimers or CPDs) and 6 − 4 photoproducts (6 − 4 PPs). Such alterations can directly or indirectly result in DNA strand breaks and contribute to the inactivation of pathogenic microorganisms [10–12]. Prior research studies has demonstrated that, contingent upon the wavelength, low doses of short-duration UVC exposure can selectively inactivate microorganisms while exerting minimal cytotoxic effects on mammalian cells [13–15]. Research indicates that the Fucoidan-UVC combination acts synergistically against oral squamous cell carcinoma cells (Ca9-22, CAL 27), inhibiting proliferation, promoting apoptosis, and causing DNA damage, while showing negligible toxicity in non-malignant oral epithelial cells, which indicates high selectivity [16]. Meanwhile, studies have shown that through systematic optimization of dose, distance, and exposure time, 275 nm UVC-LEDs can achieve rapid inactivation of SARS-CoV-2, reaching ≥ 99.9% within 10 s and exceeding 99.99% at a distance of 5 cm. This validates its efficacy as a portable disinfection solution for rapidly mitigating viral transmission risks during public health crises [17]. Based on the well-documented efficacy and established safety profile of UVC-LEDs in disinfection applications, this study proposes a novel hypothesis: can UVC irradiation be developed as an in vivo treatment for *H. pylori* infection? To test this hypothesis, we evaluated the bactericidal efficacy of UVC against *H. pylori* in vitro, as well as its safety and effectiveness within the rat stomach, aiming to investigate the clinical translational potential of this method and provide new insights for the clinical management of *H. pylori* infection.

## Methods and methods

### Animal care and ethics

Male Sprague Dawley rats (180–200 g) were acquired from the Experimental Animal Center at Xi’an Jiaotong University. The rats were housed in a specific pathogen-free environment. All animal experiments were conducted in compliance with the guidelines established by the Institutional Animal Care and Use Committee at Xi’an Jiaotong University (XJTUAE2024-2336).

### UVC light source

**Fig. 1:**
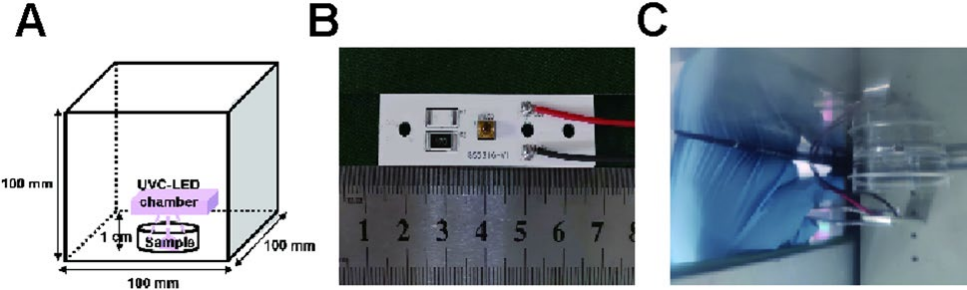
Experimental setup (**A**) Schematic of UVC-LED lamp irradiation of *H. pylori* Sydney Strain-1 (SS1); (**B**) UVC-LED lamp; (**C**) UV irradiation of *H. pylori* Sydney Strain-1 (SS1)

Three custom-built UVC-LED lamps operating at wavelengths of 254 nm, 265 nm, and 275 nm, each with a power output of 4 mW, were used in this study (Fig. 1). The lamps emit light at an angle of approximately 120°, producing an irradiance of 0.3 to 0.4 mW/cm² when measured at a distance of 1 cm from the emission window. The energy of irradiation can be regulated by modifying the duration of the irradiation process.

### Bacterial strains and culture condition

The *H. pylori* Sydney Strain-1 (SS1) was obtained from the Microbiology Laboratory within the Department of Clinical Laboratory at the First Affiliated Hospital of Xi’an Jiaotong University. Bacteria stored in culture media with 20% glycerol at − 80 °C were thawed at ambient temperature, inoculated into fresh *Helicobacter pylori* Solid Medium (HB8646, Haibo, Qingdao, China) and cultured at 37℃ under microaerophilic conditions (O_2_: 5%, CO_2_: 10%, N₂: 85%) for 3 days. Then, the colonies were harvested by centrifugation and washed with phosphate-buffered saline (PBS). The bacterial concentration was determined by plating serial dilutions and counting colony-forming units (CFU). The suspension was then diluted in fresh broth to a final density of 1 × 10^9^ CFU/ml.

### UVC irradiation to bacterial strains

Stock suspensions of bacteria were 10-fold serially diluted by sterile PBS for adjusting the bacterial load/broth to 1 × 10^5^ CFU/ml, and 330 µl bacterial broth was placed in the 35 mm petri dish. These samples were irradiated directly by UVC-LED lamps (254 nm, 265 nm, 275 nm) for durations of 0 s, 10 s, 30 s, 1 min, 3 min, 4 min, 5 min, and 10 min, with three independent replicates (*n* = 3) for each wavelength and time point. The irradiation distance was fixed at 1 cm. Post-irradiation, a 100 µl portion was harvested from the original 330 µl suspension in each dish to assess viability. This aliquot was spread plated onto *H. pylori* Solid Medium (HB8646) and incubated for 3 days under microaerophilic conditions (5% O₂, 10% CO₂, 85% N₂). Results are expressed as the inhibition percentage of bacteria compared to the control group; the experiment was repeated in triplicate. Inhibition rate was calculated using the following equation: Inhibition rate = (Control group bacterial mean − Irradiation group bacterial mean)/Control group bacterial mean ×100%.

#### Scanning electron microscopy (SEM)

Scanning electron microscopy (SEM) was employed to examine the alterations in bacterial morphology following exposure to ultraviolet (UV) light for a duration of 3 min. This specific exposure time was selected for detailed microscopic analysis based on the colony-forming unit (CFU) assay results, which indicated that it represented a critical transitional phase in the bactericidal process. At 3 min of exposure (under an irradiance of 0.3–0.4 mW/cm² and a distance of 1 cm), a significant, yet sub-lethal, reduction in viability was observed. Collected bacterial liquid after different experimental interventions in the 1.5 ml sterile centrifuge tube and centrifugated at 10 000 rpm for 2 min, discard the supernatant liquid, then the pre-cooled 2.5% glutaraldehyde was slowly added dropwise along the tube wall and fixed at 4 °C. After washed by PBS, the samples were dehydrated in the ethanol solutions with different concentrations and observed under the SME after dry coating.

### Histological and immunohistochemical analysis

To assess the degree of tissue damage, hematoxylin and eosin staining was performed. All animals were fasted for 24 h prior to surgery. Anesthesia was maintained using isoflurane (RWD, Shenzhen, China) via inhalation. The flow rates of oxygen and isoflurane were set at 0.6 L/min and 2 L/min, respectively. A 2–3 cm midline incision was made below the xiphoid process to expose the stomach. Stomach tissue was irradiated by ultraviolet (UV) light at an irradiance 0.3ཞ0.4 mW/cm^2^ for 3 min, the distance between the lamp tube and stomach tissue surface was 1 cm. Stomach specimens were dissected along the greater curvature 30 min、1 d、3 d、7 d after irradiation. After washing with physiological saline, the tissues were immediately fixed in 4% paraformaldehyde for 24 h. Paraffin-embedded blocks of Stomach specimens and 4 μm-thick paraffin sections were prepared. Then, the tissue sections were dewaxed with xylene and subjected to gradient hydration.

### DNA damage and TUNEL assay

The assessment of DNA damage resulting from UVC irradiation was conducted through immunofluorescence staining for γ-H2AX using an anti-Phospho-H2A.X (S139) rabbit polyclonal antibody (Catalog No. GB111841-50; Servicebio). Control samples comprised stomach specimens that were not subjected to irradiation. Additionally, apoptosis in the stomach specimens following UVC exposure was evaluated by terminal deoxynucleotidyl transferase-mediated nick end labelling (TUNEL) assay with a CF488 TUNEL Cell Apoptosis Detection Kit (Catalog No. G1504-50T; Servicebio). All staining procedures were performed in strict accordance with the manufacturers’ recommended protocols.

### Establishment of animal infection model and treatment of *H pylori* infection in vivo

The rats were fasted for 12 h and then subjected to a four-day bacterial inoculation regimen within the first week, receiving daily gavages of either 1 ml of saline or 1 ml of Hp strains (1 × 10^9^ CFU/ml) [18]. One week after the completion of this inoculation regimen, the rats were euthanized (Rats were euthanized by intraperitoneal injection of an overdose of sodium pentobarbital [150 mg/kg body weight]), tissue specimens were obtained from the glandular and non-glandular regions of the rat stomach through open surgery. The biopsy samples were subsequently fixed in 4% formalin, and histological analysis was conducted utilizing Giemsa staining [19]. Following the successful modeling, the rats were randomly allocated into three wavelength groups (254 nm, 265 nm, and 275 nm; *n* = 15 per group). Each group was further subdivided into five subgroups (*n* = 3) based on irradiation duration (0, 30, 60, 120, or 180 s). Prior to UVC exposure, all rats were anesthetized using isoflurane (the flow rates of oxygen and isoflurane were set at 0.6 L/min and 2 L/min, respectively) to ensure their immobility and well-being during the procedure. Seven days post-irradiation, the rats were euthanized via an intraperitoneal overdose of sodium pentobarbital (150 mg/kg), and their stomachs were harvested for analysis. The stomach was incised along the gastrointestinal tract (GIT), and the internal contents were extracted and subsequently washed with PBS. The stomachs were collected and homogenized in sterile saline at a ratio of 1:5 (weight to volume) utilizing a high-throughput tissue mill (SV48R, DHS, Tianjin, China). The resulting homogenate was subsequently plated onto selective *H. pylori* solid medium (HB8646, supplemented with 7% defibrinated sheep blood and a specific antibiotic cocktail [e.g., vancomycin, amphotericin B, etc.]). An aliquot of 100 µl was spread per plate. The plates were then incubated under microaerophilic conditions O_2_: 5%, CO_2_: 10%, N₂: 85%) for a duration of three days, after which colony counts were performed. Results are expressed as the inhibition percentage of bacteria compared to the control group; the assay was repeated in triplicate. Inhibition rate was calculated using the following equation: Inhibition rate = (Control group bacterial mean − Irradiation group bacterial mean)/Control group bacterial mean ×100%.

### Digestive function measurement

In order to assess the impact of UVC irradiation on gastric digestive function, the intragastric administration of the phenol red–methylcellulose (PR-HPMC) method was employed [20], as documented in prior research. To prepare a solution of PR-HPMC (100 mL, 50 mg/dL), 50 mg of phenol red was solubilized in a 1.5% methylcellulose medium. Gastric tissues and contents were collected immediately from euthanized rats 30 min after the intragastric administration of 2 mL PR-HPMC (Rats were euthanized by intraperitoneal injection of an overdose of sodium pentobarbital [150 mg/kg body weight]). The optical density (OD) value of the gastric supernatant was measured at a wavelength of 560 nm utilizing a microplate reader. Each data point reflects the mean value derived from three independent experiments (*n* = 3–4 wells/experiment). The processing of standard samples follows the previously outlined methodology, with the exception that the stomach and its contents were excluded from the analysis. The gastric emptying rate was calculated utilizing the formula: gastric emptying rate = (1- measured phenol red absorbance/standard phenol red absorbance) × 100%.

### Statistical analysis

Measurement data were presented as means ± standard deviations (SD). Differences were evaluated utilizing an unpaired Student’s t-test for the comparison of two data sets and one-way analysis of variance (ANOVA) followed by Post-hoc Tukey multiple comparison for three or more data sets. Categorical data were analyzed for group differences using the chi-squared test (Pearson’s chi-squared test). *P* values less than 0.05 were considered statistically significant. Data were analyzed using Prism GraphPad 8 (GraphPad Prism 8.0, La Jolla, CA, USA).

## Results

### In vitro bactericidal efficacy of UVC irradiation against *H. pylori*

**Fig. 2:**
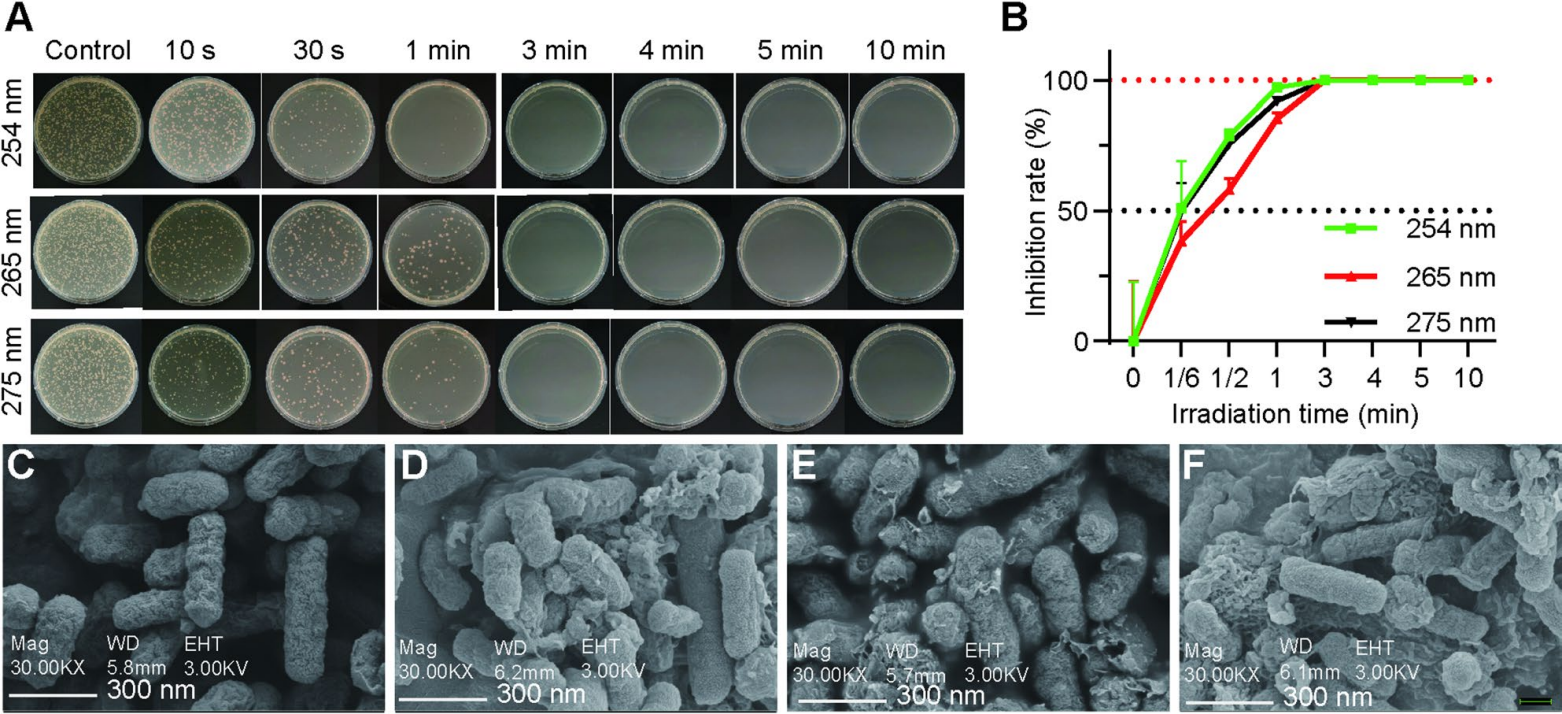
Bactericidal effect by the UVC-LED (254 nm, 265 nm, and 275 nm) light in vitro (*n* = 3). **A** The SS1 was irradiated with the UVC-LED (254 nm, 265 nm, and 275 nm) light for 0, 10 s, 30 s, 1 min, 3 min, 4 min, 5–10 min with a power density of 0.3ཞ0.4 mW/cm^2^. **B** Time course bacterial inhibition rates after irradiated with the UVC-LED (254 nm, 265 nm, and 275 nm) light for different times. *H. pylori* Sydney Strain-1 before (**C**) and after UV irradiation at 254 nm (**D**), 265 nm (**E**), and 275 nm (**F**) with a power density of 0.3ཞ0.4 mW/cm^2^, as shown SEM. SEM images (30.00 KX magnification), scale bars represent 300 nm

The alterations in the growth of the SS1 strain under the influence of ultraviolet light at three distinct wavelengths (254 nm, 265 nm, and 275 nm) are illustrated in Fig. 2A. The experimental findings indicated that an increase in irradiation duration leads to enhanced disinfection efficacy in the irradiated groups when compared to the non-irradiated group. At 30-second exposure, ANOVA revealed significant differences among wavelengths (F(2,6) = 102.35, *p* < 0.0001). Post-hoc tests showed that 254 nm and 275 nm provided comparable inhibition (*p* = 0.142), with both being significantly more effective than 265 nm (*p* < 0.001 for both comparisons), and the inhibition rates for ultraviolet light at wavelengths of 254 nm, 265 nm, and 275 nm were recorded at 79%, 58%, and 75%. After one minute, these rates increased to 97%, 85%, and 92%, one-way ANOVA revealed significant overall differences among the three wavelengths (F(2,6) = 87.42, *p* < 0.0001). Post-hoc Tukey HSD tests confirmed that 254 nm produced significantly greater inhibition compared to 265 nm (mean difference = 11.93, *p* = 0.0008, Cohen’s d = 6.23, 95% CI [2.45, 10.01]) and 275 nm (mean difference = 5.14, *p* = 0.022, Cohen’s d = 4.82, 95% CI [1.78, 7.86]), as illustrated in Fig. 2B. The findings indicate that at 1-minute exposure time, the 254 nm wavelength was the most effective. However, for longer exposure times (3–10 min), all three wavelengths performed equally well, achieving complete or near-complete disinfection.

### SEM analysis of *H. pylori* SS1 before and after exposure to UVC irradiation

Under electron microscopy, *H. pylori* that has not been subjected to UV irradiation exhibited a unipolar morphology characterized by multiple flagella, along with bluntly rounded ends and a spirally curved surface that remained intact (Fig. 2C). Following exposure to UV light at wavelengths of 254 nm, 265 nm, and 275 nm, significant damage was noted on the cell surface, accompanied by morphological alterations. This suggests that irradiation at all three wavelengths resulted in detrimental effects on the cell membrane of *H. pylori*, as illustrated in Fig. 2 (D, E, F).

### Histopathology

**Fig. 3:**
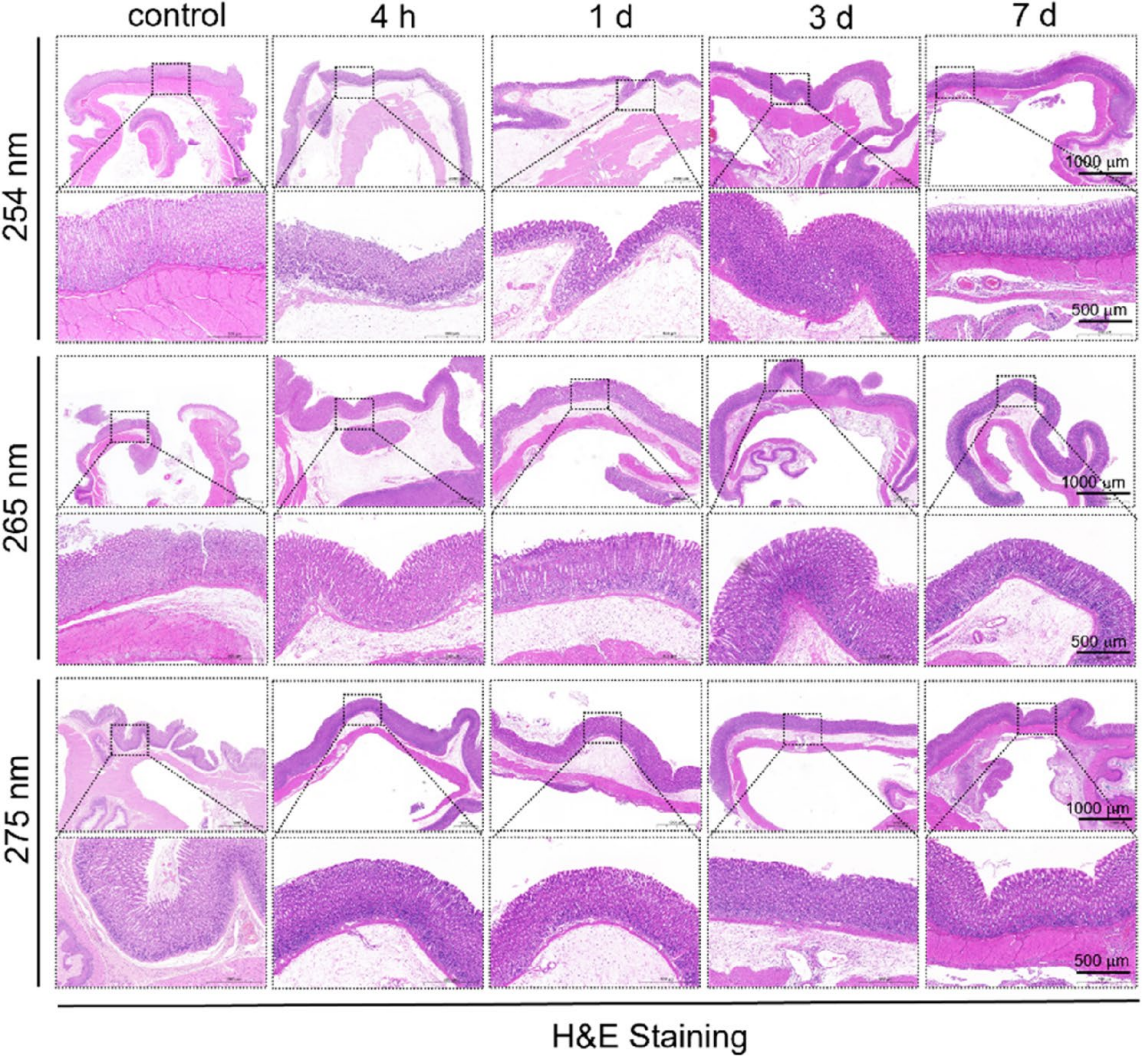
Histopathology of the rat gastric mucosa before and after UV irradiation at 254 nm, 265 nm, and 275 nm with an irradiance 0.3ཞ0.4 mW/cm^2^ for 3 min. H&E staining of the rat gastric mucosa from control and irradiated 254 nm rats at 4 h to 7 d. H&E staining of the rat gastric mucosa from control and irradiated 265 nm rats at 4 h to 7 d. H&E staining of the rat gastric mucosa from control and irradiated 275 nm rats at 4 h to 7 d. Scale bars represent 500 μm

Figure 3 illustrates the hematoxylin and eosin (H&E) staining of the gastric wall at 7 days following irradiation. The findings indicate that the mucosal epithelial cells remained intact and exhibited only light staining. Additionally, the gastric foveolar architecture was not significantly compromised, and the gastric gland cavities within the lamina propria appeared to be preserved. The structure of the mucosal muscularis also remained intact, showing no notable alterations. These observations suggest that there were no significant differences in the gastric wall within four hours following exposure to UVC irradiation. After a one-day exposure to UVC radiation, edema was noted, and infiltration of inflammatory cells was observed within the mucosal layer. Three days post-irradiation, a significant decrease in mucosal edema was observed, along with a slight infiltration of red blood cells. By the seventh day after irradiation, the regeneration of the gastric wall showed no substantial differences when compared to the control group. The experimental results demonstrated a robust relationship between the extent of mucosal injury to the gastric wall and the wavelength of irradiation. Notably, the severity of damage recorded in the groups subjected to wavelengths of 254 nm and 265 nm was significantly more pronounced than the group of 275 nm. This indicates that the gastric mucosa demonstrates safety when exposed to low doses of 275 nm irradiation, and that any resultant tissue damage can be repaired within a relatively brief timeframe.

#### DNA damage and cellular apoptosis induced by UVC irradiation in rat gastric tissue

**Fig. 4:**
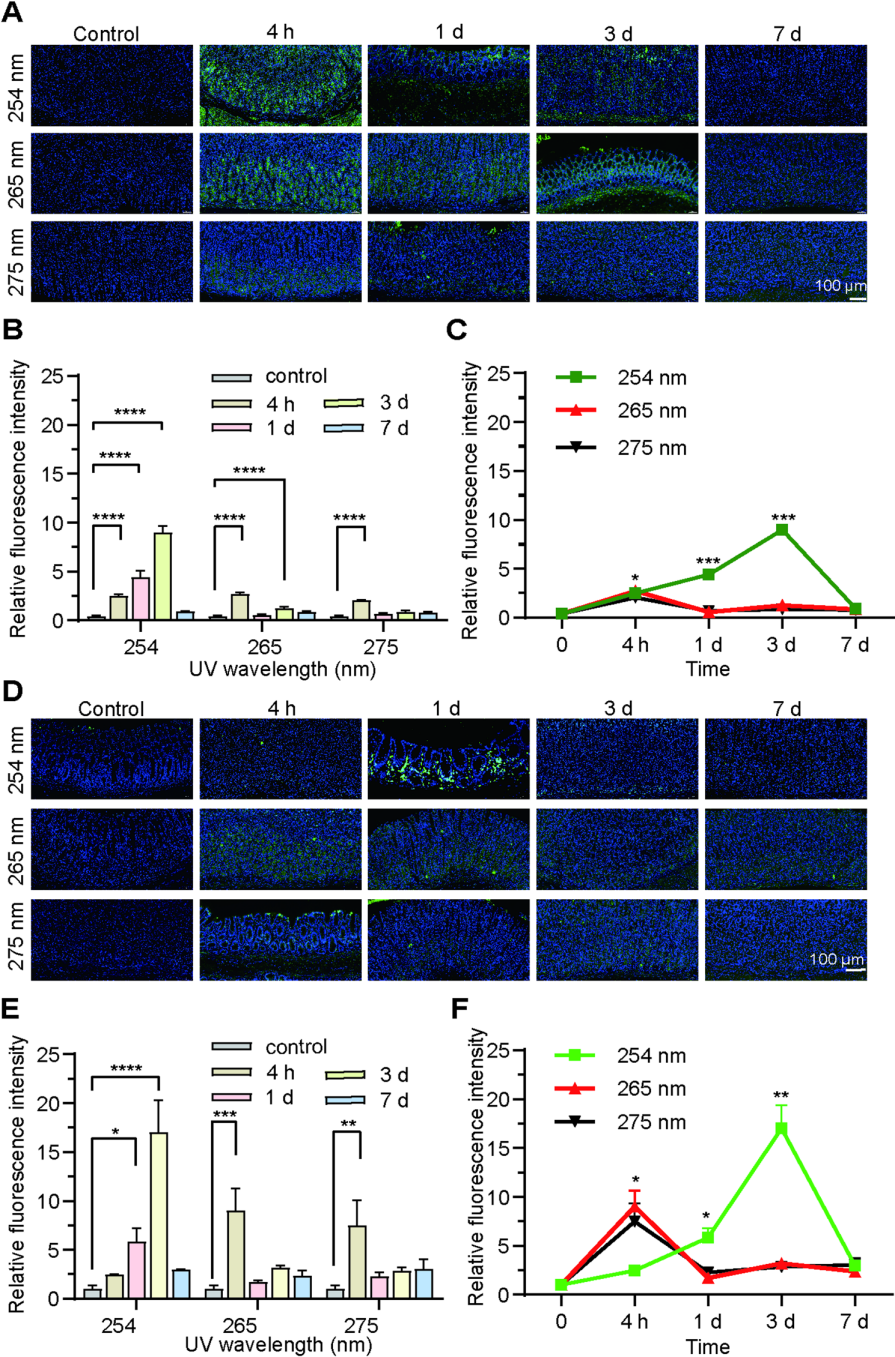
**A** Representative immunofluorescence images of the gastric wall before and after UV irradiation at 254 nm, 265 nm, and 275 nm with an irradiance 0.3ཞ0.4 mW/cm^2^ for 3 min. Representative immunofluorescence images of the gastric wall from control and irradiated 254 nm rats at 4 h to 7 d. Representative immunofluorescence images of the gastric wall from control and irradiated 265 nm rats at 4 h to 7 d. Representative immunofluorescence images of the gastric wall from control and irradiated 275 nm rats at 4 h to 7 d. Scale bars represent 50 μm. **B**, **C** Quantitative assessment of relative fluorescence intensity of γ-H2AX in the gastric wall before and after UV irradiation at 254 nm, 265 nm, and 275 nm with an irradiance 0.3ཞ0.4 mW/cm^2^ for 3 min. ∗∗ *p* < 0.01 and ∗∗∗∗ *p* < 0.0001 compared with the control. **D** TUNEL staining of the gastric wall before and after UV irradiation at 254 nm, 265 nm, and 275 nm with an irradiance 0.3ཞ0.4 mW/cm^2^ for 3 min. Scale bars represent 50 μm. (**E**, **F**) Quantitative assessment of relative fluorescence intensity in the gastric wall before and after UV irradiation at 254 nm, 265 nm, and 275 nm with an irradiance 0.3ཞ0.4 mW/cm^2^ for 3 min. ∗ *p* < 0.05, ∗∗*p* < 0.01, ∗∗∗*P*<0.001 and ∗∗∗∗*P*<0.0001 compared with the control

Figure 4A presentative images of the gastric wall, wherein the cell nuclei, which are stained blue with DAPI, exhibit green fluorescence corresponding to γ-H2AX following 3 min of UVC irradiation at wavelengths of 254 nm, 265 nm, and 275 nm. In the untreated rat, which served as the negative control, minimal levels of γ-H2AX were detected within the cell nuclei. Statistical analysis of DNA damage percentages across the three wavelengths revealed highly significant effects of both time (F(4,24) = 89.45, *p* < 0.0001) and wavelength (F(2,6) = 85.73, *p* < 0.0001), with a significant time × wavelength interaction (F(8,24) = 42.18, *p* < 0.0001). Post-hoc analyses identified that 254 nm wavelength produced significantly greater DNA damage compared to both 265 nm and 275 nm at the 1-day (*p* < 0.0001) and 3-day (*p* < 0.0001) timepoints, with large effect sizes (ηp² >0.97). However, after a period of 7 days, no statistically significant difference was observed between the irradiation group and the control group (*P* > 0.5) Fig. 4 (B, C). These findings demonstrate wavelength-specific DNA damage kinetics, with 254 nm inducing a more pronounced but transient DNA damage response compared to the other wavelengths tested.

The TUNEL assay was conducted to assess the levels of apoptosis in rat gastric tissues (Fig. 4D). Statistical analysis of TUNEL assay results across the three wavelengths revealed highly significant effects of time (F(4,12) = 25.73, *p* < 0.0001), wavelength (F(2,3) = 28.95, *p* = 0.009), and their interaction (F(8,12) = 15.42, *p* < 0.0001). Post-hoc analyses demonstrated that 254 nm wavelength induced a distinct biphasic response: it initially showed significantly lower TUNEL positivity at 4 h compared to other wavelengths (*p* < 0.05), but subsequently produced significantly higher apoptosis/DNA damage at 1-day (*p* < 0.05) and 3-day (*p* < 0.01) timepoints, with very large effect sizes (ηp² >0.94). By 7 days, all wavelengths returned to comparable baseline levels (*p* = 0.493) Fig. 4 (E, F). These findings demonstrate that UVC irradiation triggers a timed apoptotic response: TUNEL-positive cells increased and migrated into the mucosal muscular layer by day 1, became diffusely distributed by day 3, and subsided by day 7. This resolution, coupled with the lack of significant difference from the control group at day 7, indicates active tissue self-repair. Critically, statistical analyses reveal that the 275 nm wavelength induced a significantly lower apoptotic response compared to 254 nm at the critical 1-day (*p* < 0.05) and 3-day (*p* < 0.01) timepoints, underscoring its more favorable safety profile among the wavelengths tested.

### *H. pylori* Eradication by UV irradiation

**Fig. 5:**
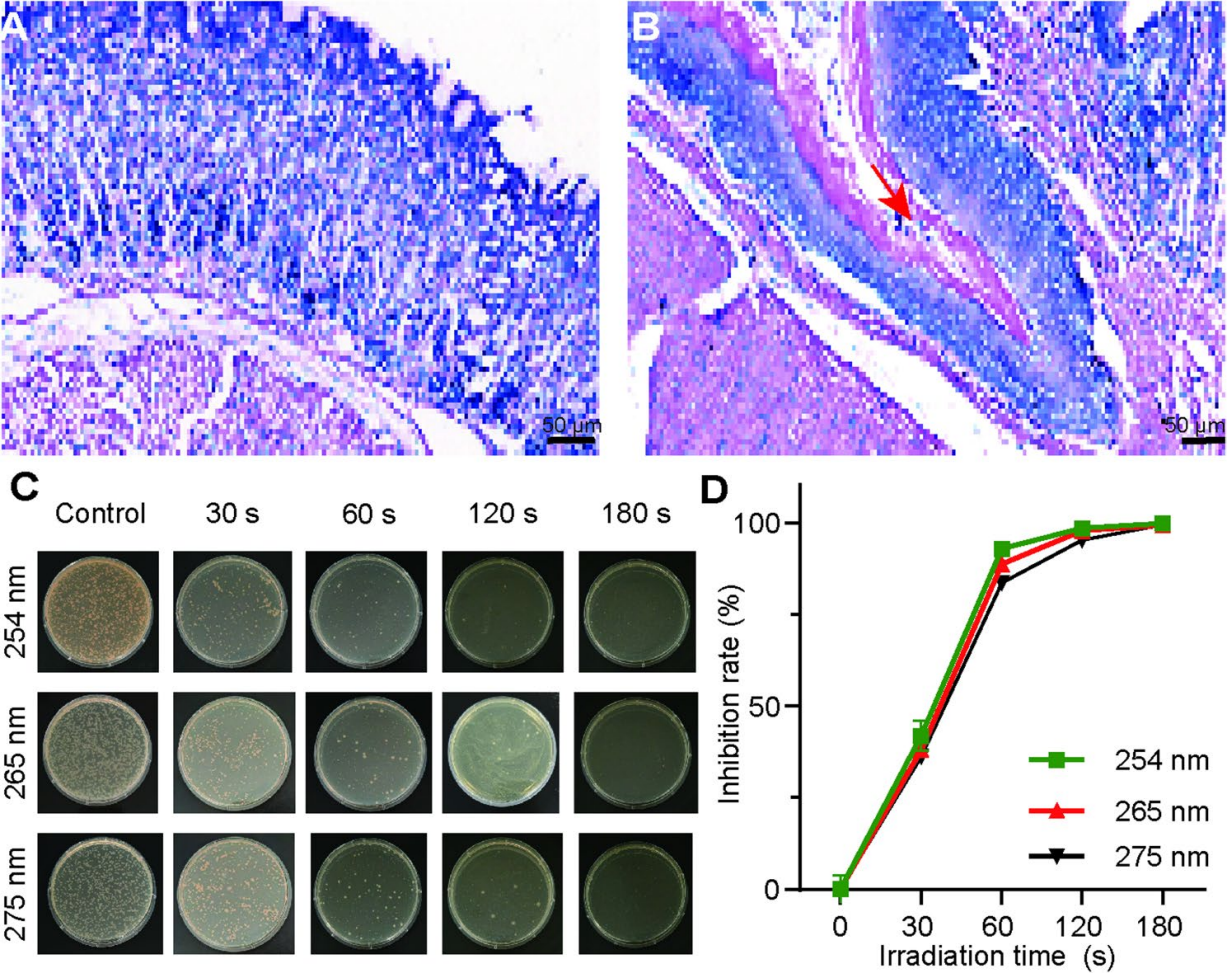
Giemsa staining of gastric tissue of normal rats and *H. pylori* model rats. **A** Gastric tissue of normal rats. **B** Gastric tissue of Hp model rats, the bacteria are present in the mucosal layer, Hp is blue (red arrows), short rod-shaped, or “S” shaped. Scale bars represent 50 μm. **C** Bactericidal effect and influence by the UVC-LED (254 nm, 265 nm, and 275 nm) light on the Hp infected of rat stomach tissues. The infected stomach tissues of the rats was treated with the UVC-LED (254 nm, 265 nm, and 275 nm) light for 0, 30 s, 60 s, 120 s, or 180 s with a power density of 0.3–0.4 mW/cm^2^. **D** Time course bacterial inhibition rates in the Hp infected of rat stomach tissues after treatment with the UVC-LED (254 nm, 265 nm, and 275 nm) light for different times

Rats were euthanized within one week following the final bacterial inoculation, and gastric mucosal tissue specimens were collected for culture and Giemsa staining. Subsequent to isolation and culture, the rats exhibited a positive status for *H. pylori*, characterized by extensive colonization of the gastric mucosa and gastric pits. Figure 5A illustrates the Giemsa staining of gastric tissue from normal rats, while Fig. 5B depicts the gastric tissue of *H. pylori* model rats, wherein *H. pylori* is indicated in blue (as denoted by red arrows) and appears in short rod-shaped or “S” shaped forms. Following the establishment of the model, the rats underwent treatment with UVC irradiation at wavelengths of 254 nm, 265 nm, and 275 nm. The gastric mucosal tissue was subsequently collected on days 7 to 14 post-treatment. The colonization of *H. pylori* in the stomach was assessed through quantitative culture methods. As illustrated in Fig. 5 (C, D), one-way ANOVA confirmed significant overall differences (F(2,6) = 31.72, *p*<0.01). Post-hoc Tukey HSD tests revealed that 254 nm produced significantly greater inhibition compared to both 265 nm (mean difference = 4.19, *p* = 0.049, Cohen’s d = 2.38, 95% CI [0.01, 4.75]) and 275 nm (mean difference = 9.30, *p* = 0.001, Cohen’s d = 5.10, 95% CI [1.71, 8.49]), but the survival rate of Hp in the UVC treatment group was markedly lower in comparison to the control group.

#### Digestive function of the stomach

**Fig. 6:**
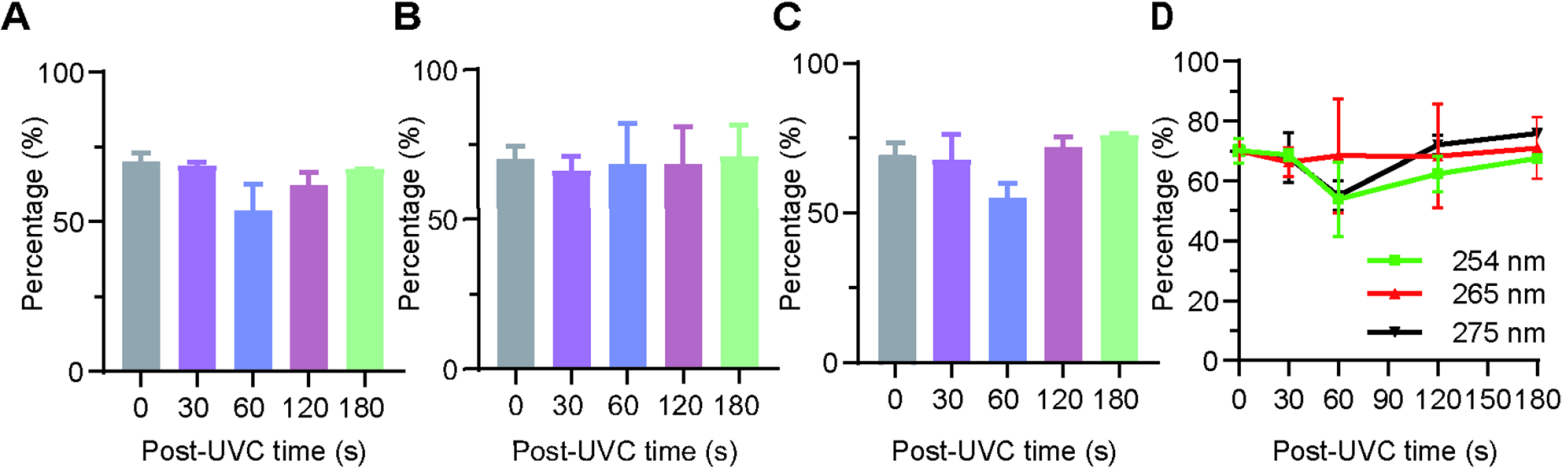
Changes in digestive function post-UVC irradiation. **A**, **B** and **C** The gastric emptying rates of the rats was measured before and after irradiated by the UVC-LED (254 nm, 265 nm, and 275 nm) light for 0, 30 s, 60 s, 120 s, or 180 s with a power density of 0.3–0.4 mW/cm^2^ (*p* > 0.05). **D** Time-dependent impacts of 254 nm, 265 nm, and 275 nm light exposure on gastric function percentages

Figure 6 illustrates the changes in digestive function observed in rats subsequent to exposure to irradiation. The gastric emptying rate serves as a significant parameter for assessing the gastric motility function within the body. Consequently, this study employs the PR-HPMC method to evaluate the gastric emptying rate. As illustrated in Fig. 6 (A, B, C), at that specific time point, no statistically significant difference was observed between the test group and the control group (*p* > 0.05). As illustrated in Fig. 6 (D), the analysis revealed a significant main effect of time (F (4,12) = 8.32, *p* = 0.002, ηp² = 0.735) and a significant time × wavelength interaction (F (8,12) = 3.45, *p* = 0.028, ηp² = 0.697). However, no significant main effect of wavelength was found (F (2,3) = 0.84, *p* = 0.512). Simple effects analysis at each time point showed no statistically significant differences between wavelengths at any specific exposure duration (all *p* > 0.05), though visual inspection of the data suggests some time-dependent variation in the response patterns across wavelengths.

## Discussion

*H. pylori* is significantly linked to several chronic conditions, including chronic superficial gastritis, chronic atrophic gastritis and peptic ulcer disease [21, 22]. The prolonged coexistence of *H. pylori* with patients, along with its significant association with a range of diseases, underscores the imperative need for the eradication of *H. pylori*. The existing guidelines suggest that quadruple therapy, which includes a proton pump inhibitor (PPI), clarithromycin, amoxicillin, and bismuth, is the preferred first-line treatment for *H. pylori* infection [23, 24]. However, the efficacy of quadruple therapy is often limited due to antibiotic resistance, which has given rise to many other therapies, such as vaccination, vitamin and garlic diet therapy [25], etc., but none of them can completely eradicate *H. pylori*. In comparison to conventional chemotherapy, UVC irradiation therapy exhibits a wider antibacterial spectrum and is less likely to induce drug resistance [26].

UVC irradiation presents a promising approach as a novel antibacterial method for the treatment of *H. pylori* infections. Nonetheless, additional research concentrating on safety and ethical considerations must be undertaken prior to the potential implementation of clinical applications. In this investigation, we have demonstrated that UVC irradiation is capable of effectively eliminating gastric *H. pylori*. Simultaneously, normal rats were subjected to UVC irradiation. The findings indicate that, in comparison to 275 nm, the wavelengths of 254 nm and 265 nm can inflict significant damage to the gastric mucosal layer; however, this damage appears to revert to baseline levels after a period of seven days. Gastric motility remains unaffected following UVC intervention. This research will contribute to future investigations and clinical applications in the treatment of *H. pylori* by offering evidence regarding the safety and feasibility of utilizing rat models in both in vitro and in vivo studies.

It is widely recognized that UVC possesses significant disinfectant properties. The findings of our research indicate that ultraviolet light at a wavelength of 254 nm exhibits superior disinfection efficacy compared to wavelengths of 265 nm and 275 nm in the inactivation of *H. pylori*. A research study has examined the impact of 254 nm UVC radiation on the disinfection of *Bacillus lipolyticus*. The findings indicated a significant decrease in the spore count of the bacteria subjected to UVC irradiation [27]. Given the existing body of research, this study demonstrated that UVC intragastric irradiation is effective in eliminating this pathogen. In order to explore an effective strategy for the eradication of intragastric *H. pylori* and to diminish the recurrence rate of *H. pylori* infection, innovative micro/nanomaterial carriers have been engineered to enhance the efficacy of targeted UVC delivery [28]. UVC light with capsule endoscopy and applying UVC irradiation to the targeted lesion may represent a promising avenue for enhancing current deep ultraviolet disinfection techniques [29].

“Safe UVC” refers to a spectrum of ultraviolet radiation that effectively eliminates over 99% of bacteria while minimizing harm to host cells. This form of irradiation penetrates the cell membrane and inflicts damage on the DNA located within the nucleus [13, 30]. We employed various wavelengths of ultraviolet C (UVC) light, specifically 254 nm, 265 nm, and 275 nm, alongside unirradiated controls for comparative analysis. Within 0–3 days after 3 min of irradiation at wavelengths of 254 nm, 265 nm, and 275 nm, DNA damage was observed in nearly all cell nuclei. Notably, the extent of damage in the group exposed to 254 nm irradiation was more pronounced compared to the groups subjected to 265 nm and 275 nm irradiation. There was no significant difference between the three experimental groups and the control group after 7 days. Research has demonstrated that CPD induced by UVC radiation in biological tissues can be repaired within a relatively brief period. An in vivo investigation revealed that exposure to approximately 25 mJ/cm² of 254 nm UVC radiation resulted in DNA damage within the wounds of mice. However, it was observed that this DNA damage underwent significant repair within a 48-hour period [31]. A separate investigation indicates that 275 nm UVC-LED effectively inactivates bacteria and fungi in vitro within a brief exposure duration, while also demonstrating no detrimental effects on DNA or epidermal integrity in murine skin [15]. Furthermore, the preclinical safety and efficacy of low-dose UVC for the treatment of corneal infections was corroborated through studies conducted on nude mice [32, 33]. This discovery suggests the possibility of identifying a specific safe dosage of UVC irradiation that can effectively inactivate bacteria, fungi, and viruses.

### Limitations and perspectives for clinical translation

While our study provides compelling evidence for the short-term safety of 275 nm UVC irradiation, particularly its superior profile in minimizing acute DNA damage and apoptosis compared to other wavelengths, we acknowledge the valid concern regarding long-term safety and potential carcinogenic risk. The current investigation was designed to evaluate the acute therapeutic efficacy and immediate tissue responses. As such, the experimental timeline did not encompass a long-term follow-up period to assess latent oncogenic transformations.

The carcinogenic risk of UV radiation is well-documented, primarily associated with cumulative DNA damage and mutations. However, several factors specific to our intervention may mitigate this risk in a clinical context. First, targeted and localized application [13], unlike environmental UV exposure, the proposed UVC therapy is highly localized, delivered directly to the gastric mucosa via endoscope for a brief, therapeutic duration. This minimizes exposure to non-target tissues. Second, dose optimization and pulsing [9], our findings that 275 nm causes significantly less γ-H2AX foci formation (a marker of DNA double-strand breaks) suggest a reduced genotoxic potential. Future clinical protocols can be further optimized by employing pulsed UVC delivery rather than continuous wave, allowing for cellular repair intervals, and by determining the minimum bactericidal dose to limit total energy exposure. Third, wavelength-dependent safety, the 275 nm wavelength appears to reside in a “therapeutic window,” balancing microbial absorption for lethality and limited tissue penetration depth. This inherently restricts its interaction with the proliferative stem cell compartment located deeper in the gastric glands, which is crucial for long-term tissue homeostasis and cancer risk.

To conclusively address the long-term safety and therapeutic efficacy, future work must include: Long-term animal studies tracking subjects for 6–12 months post-treatment to monitor for any pathological changes, including pre-neoplastic lesions, and to rigorously assess *H. pylori* eradication status using multiplex real-time PCR on serial gastric mucosal biopsies [34]. Comprehensive genotoxicity assessments using more sensitive assays (e.g., comet assay, micronucleus test) in relevant in vitro 3D models or in vivo [35]. Investigations into DNA repair mechanisms post-UVC exposure, to determine if the minimal damage induced by 275 nm is efficiently repaired by the mucosal cells. In vivo microbiome analysis, utilizing animal models (e.g., *H. pylori*-infected mice) to assess the short- and long-term effects of gastric UVC exposure on the composition and diversity of the gastric and intestinal microbiota through 16 S rRNA sequencing. Increase the sample size to detect potential interactions between wavelength and time.

In conclusion, while the definitive exclusion of long-term risk requires further investigation, our data strongly indicate that 275 nm UVC presents a markedly lower potential for genotoxicity compared to conventional UVC wavelengths. It represents a promising candidate for a localized, non-antibiotic therapy, and its risk-benefit profile—particularly for eradicating antibiotic-resistant *H. pylori*—appears highly favorable. The next critical step towards clinical translation will be the rigorous evaluation of its long-term safety in advanced models.

## Conclusions

This work is the first empirical investigation of the antibacterial efficacy and mucosal safety of three UVC wavelengths (254 nm, 265 nm, and 275 nm) against *H. pylori* in both in vitro and in vivo models. Our findings confirm the efficacy of UVC irradiation in inactivating *H. pylori* within the gastric environment of rats. Wavelengths of 275 nm demonstrated a markedly reduced capacity to induce DNA damage and apoptosis in the gastric mucosa, and moreover, this wavelength treatment did not adversely affect gastric motility in rats. This research substantiates that the 275 nm UVC represents a promising non-antibiotic targeted therapeutic strategy against *H. pylori*, providing valuable insights for future clinical applications.

## Data Availability

The datasets used and/or analysed during the current study are available from the corresponding author on reasonable request.

## Abbreviations

UVC: Ultraviolet C
HP: Helicobacter pylori
HE: Hematoxylin and eosin
TUNEL: Terminal dUTP Nick End Labeling
MALT: Mucosa-associated lymphoid tissue
SS1: The H. pylori Sydney Strain-1
PBS: Phosphate-buffered saline
SEM: Scanning electron microscopy
PR-HPMC: Phenol red–methylcellulose
